# Survival rates are higher in married patients with biliary tract cancer: a population-based study

**DOI:** 10.18632/oncotarget.24170

**Published:** 2018-01-11

**Authors:** Wei Song, Dong-Liu Miao, Lei Chen

**Affiliations:** ^1^ Department of Intervention and Vascular Surgery, Affiliated Suzhou Hospital of Nanjing Medical University, Suzhou Municipal Hospital, Suzhou Cancer Medical Center, Suzhou, China

**Keywords:** biliary tract cancer, marital status, SEER, survival analysis, prognosis

## Abstract

Marital status has been identified as a prognostic factor in multiple malignancies. In this study, we assessed the prognostic value of marital status in 24,035 patients from the Surveillance, Epidemiology, and End Results database diagnosed with biliary tract cancer (BTC) between 2004 and 2014. Widowed patients were more likely to be women, elderly (> 60 years), have gallbladder cancer, and have localized SEER Stage disease than all other patients. Marital status was identified as an independent prognostic factor in both univariate and multivariate analyses, and cause-specific survival (CSS) rates were higher in married patients than unmarried patients. In addition, CSS rates were higher in ampulla of Vater cancer patients than in gallbladder cancer or cholangiocarcinoma patients. Further analysis revealed that CSS rates were lowest in widowed patients at each TNM stage and for all tumor sites. These results suggest marital status is a prognostic factor for clinical outcomes in patients with BTC, and widowed patients are at greater risk of cancer-specific mortality.

## INTRODUCTION

Biliary tract cancer (BTC) is a category of tumors that includes gallbladder cancer (GBC), cholangiocarcinoma, and ampullary cancer [1, 2]. Although BTC is rare, its incidence is increasing worldwide [3, 4]. In Japan, BTC is the sixth leading cause of cancer-related death, and more than 18,000 patients die from the disease annually [5]. Radical resection remains the only curative treatment for BTC, and recurrence rates after resection are high [6, 7]. Moreover, because BTC is usually diagnosed at an advanced stage, most patients are not considered candidates for curative resection. Despite recent improvements in surgical techniques and adjuvant therapy, the overall prognosis of BTC remains poor [8, 9]. It is therefore vital to identify factors that predict prognosis in BTC patients to help clinicians implement better therapeutic strategies.

Studies have identified a positive correlation between marital status and survival in BTC patients, and married people are healthier overall and live longer than unmarried people (divorced, separated, never married) [10–12]. In a large study of multiple cancer treatment sites, married patients were more likely to present with early stage disease upon diagnosis and to receive definitive treatment compared to unmarried patients [13]. Being married is also positively associated with overall and cancer-related survival for various types of cancer, such as hepatocellular carcinoma [14], breast cancer [15], gastric adenocarcinoma [16], and pancreatic cancer [17]. According to a larger-scale population-based study of information from the Surveillance, Epidemiology, and End Results (SEER) database, unmarried patients are at significantly higher risk of presentation with metastatic cancer, undertreatment, and cancer-related death [13]. However, little is known about the association between marital status and BTC. In this study, we investigated the association between marital status and cause-specific survival (CSS) in BTC patients by examining data from the Surveillance, Epidemiology, and End Results (SEER) cancer registry program for individuals diagnosed between 2004 and 2014.

## MATERIALS AND METHODS

### Patient population and study design

The Surveillance, Epidemiology, and End Results (SEER) Program of the US National Cancer Institute (NCI) was used as the data source for this population-based investigation. The SEER Program captures approximately 97% of incident cancers, and the 17 SEER tumor registries encompass approximately 28% of the US population [18]. The SEER Program collects information on cancer incidence, prevalence, survival, and cancer patient mortality.

We used the SEER Program to identify 24035 patients who were diagnosed with biliary tract cancer between 2004 and 2014. Patients with a diagnosis of biliary tract cancer (International Classification of Diseases for Oncology, Third Edition [ICD-O-3], histology codes 8010, 8020, 8040, 8041, 8070, 8140, 8144, 8160, 8161, 8162, 8260, 8310, 8480, 8490, and 8560, and site codes C23.0, C23.9, C22.1, C24.0, and C24.1) were considered for analysis. Patients more than 18 years old for whom marital status, cause of death, and survival duration in months were known were included in the current study. Patients with multiple primary cancers were excluded if BTC was not the first disease diagnosed.

This study was based on public data from the SEER database; we obtained permission to access research data files (reference number 10091-Nov 2016). The data did not include the use of human subjects or personal identifying information; thus, informed consent was not required.

### Study variables

Several variables, including demographics (sex, age, race), TNM stage, extent of disease (localized, regional, distant), tumor site (gallbladder cancer and cholangiocarcinoma, ampulla of Vater cancer), tumor grade (well-differentiated, moderately differentiated, poorly differentiated, undifferentiated, unknown), histologic type, treatment, and marital status at the time of diagnosis were examined. Marital status, the major variable of interest, was classified in a binary manner (married vs unmarried). The unmarried category included widowed and separated/divorced patients as well as those who never married. The AJCC 6th TNM staging system was used; because this staging system became publicly available in 2004, we restricted our study to patients diagnosed from 2004–2014. According to the SEER staging system, tumors that remain *in situ*, or confined to the organ of origin, were considered localized. Those with local invasion or metastasis to regional lymph nodes were categorized as regional, while those with cancer that traveled to distant organs were categorized as distant.

### Statistical analyses

Baseline patient demographics and disease characteristics were compared using *t*-tests or chi-square tests as appropriate. The Kaplan-Meier method was used to assess survival functions. The log rank test was used to test differences between survival curves. Cox proportional hazards multivariable regression was used to assess the impact of marital status on cancer-specific mortality. The primary outcomes of interest in this study was 5-year CSS, which was calculated from the date of diagnosis to the date of cancer-specific death. Deaths attributed to BTC were treated as events, while deaths from other causes were treated as censored observations. All *P* values were 2-sided, and *P* values < 0.05 were considered statistically significant. All statistical analyses were performed using SPSS version 23 (Statistics Package for Social Science, Chicago, IL).

## RESULTS

### Patient characteristics

A total of 24035 qualified patients who were diagnosed during the 10-year study period (2004 to 2014) were identified. Of these patients, 10774 (44.8%) were male and 13261 (55.2%) were female. 13268 (55.2%) were married, 5062 (21.1%) were widowed, 3347 (13.9%) had never married, and 2358 (9.8%) were divorced or separated. Higher proportions of patients in the widowed group were women, elderly (> 60 years), had gallbladder cancer, and had more localized SEER Stage disease compared to all other patients (all *p* < 0.001). Patient demographics and pathological features are shown in Table 1.

**Table 1:** Baseline demographic and tumor characteristics of SEER database patients

	Total	Married	Widowed	Never married	Divorced/ Separated	*P*
Characteristic	(*n* = 24035)	(*n* = 13268)	(*n* = 5062)	(*n* = 3347)	(*n* = 2358)	
	*N*	*N* (%)	*N* (%)	*N* (%)	*N* (%)	
Sex						< 0.001
Male	10774	7396 (55.7)	898 (17.7)	1530 (45.7)	950 (40.3)	
Female	13261	5872 (44.3)	4164 (82.3)	1817 (54.3)	1408 (59.7)	
Age						< 0.001
≤ 60	6314	3866 (29.1)	210 (4.1)	1452 (43.4)	786 (33.3)	
> 60	17721	9402 (70.9)	4852 (95.9)	1895 (56.6)	1572 (66.7)	
Race						< 0.001
White	18478	10302 (77.6)	3935 (76.4)	2405 (73.8)	1836 (77.9)	
Black	2270	886 (6.7)	579 (11.2)	489 (15.0)	316 (13.4)	
Other^*^	3287	2080 (15.7)	638 (12.4)	363 (11.1)	206 (8.7)	
Tumor site						< 0.001
Gallbladder	7869	4014 (30.3)	1938 (38.3)	1120 (33.5)	797 (33.8)	
Bile duct	12668	7176 (54.1)	2503 (49.4)	1748 (52.2)	1241 (52.6)	
Ampulla of Vater	3498	2078 (15.7)	621 (12.3)	479 (14.3)	320 (13.6)	
AJCC 6th TNM stage						< 0.001
I	5205	2842 (22.2)	1224 (22.0)	675 (20.2)	464 (19.7)	
II	5109	2974 (23.3)	938 (16.9)	690 (20.6)	507 (21.5)	
III	2715	1627 (12.7)	432 (7.8)	356 (10.6)	300 (12.7)	
IV	7521	4239 (33.2)	1373 (24.7)	1117 (33.4)	792 (33.6)	
Unknown	3485	1095 (8.6)	1586 (28.6)	509 (15.2)	295 (12.5)	
Grade						< 0.001
I	1702	985 (7.4)	334 (6.6)	237 (7.1)	146 (6.2)	
II	5891	3443 (25.9)	1059 (20.9)	819 (24.5)	570 (24.2)	
III	5139	3006 (22.7)	943 (18.6)	682 (20.4)	508 (21.5)	
IV	220	125 (0.9)	52 (1.0)	26 (0.8)	17 (0.7)	
Unknown	11083	5709 (43.0)	2674 (52.8)	1583 (47.3)	1117 (47.4)	
SEER Stage						< 0.001
Localized	5526	2870 (21.6)	1390 (27.5)	757 (22.6)	509 (21.6)	
Regional	7853	4680 (35.3)	1370 (27.1)	1051 (31.4)	752 (31.9)	
Distant	8367	4743 (35.7)	1504 (29.7)	1227 (36.7)	893 (37.9)	
Unknown	2289	975 (7.3)	798 (15.8)	312 (9.3)	204 (8.7)	
Therapy						< 0.001
Surgery	10169	6046 (45.6)	1788 (35.3)	1353 (40.4)	982 (41.6)	
No surgery	13629	7107 (53.6)	3207 (63.4)	1962 (58.6)	1353 (57.4)	
Unknown	237	115 (0.9)	67 (1.3)	32 (1.0)	23 (1.0)	

^*^: Other includes American Indian/Alaska native, Asian/Pacific Islander, and unknown.

### Effect of marital status on CSS

**Figure 1:**
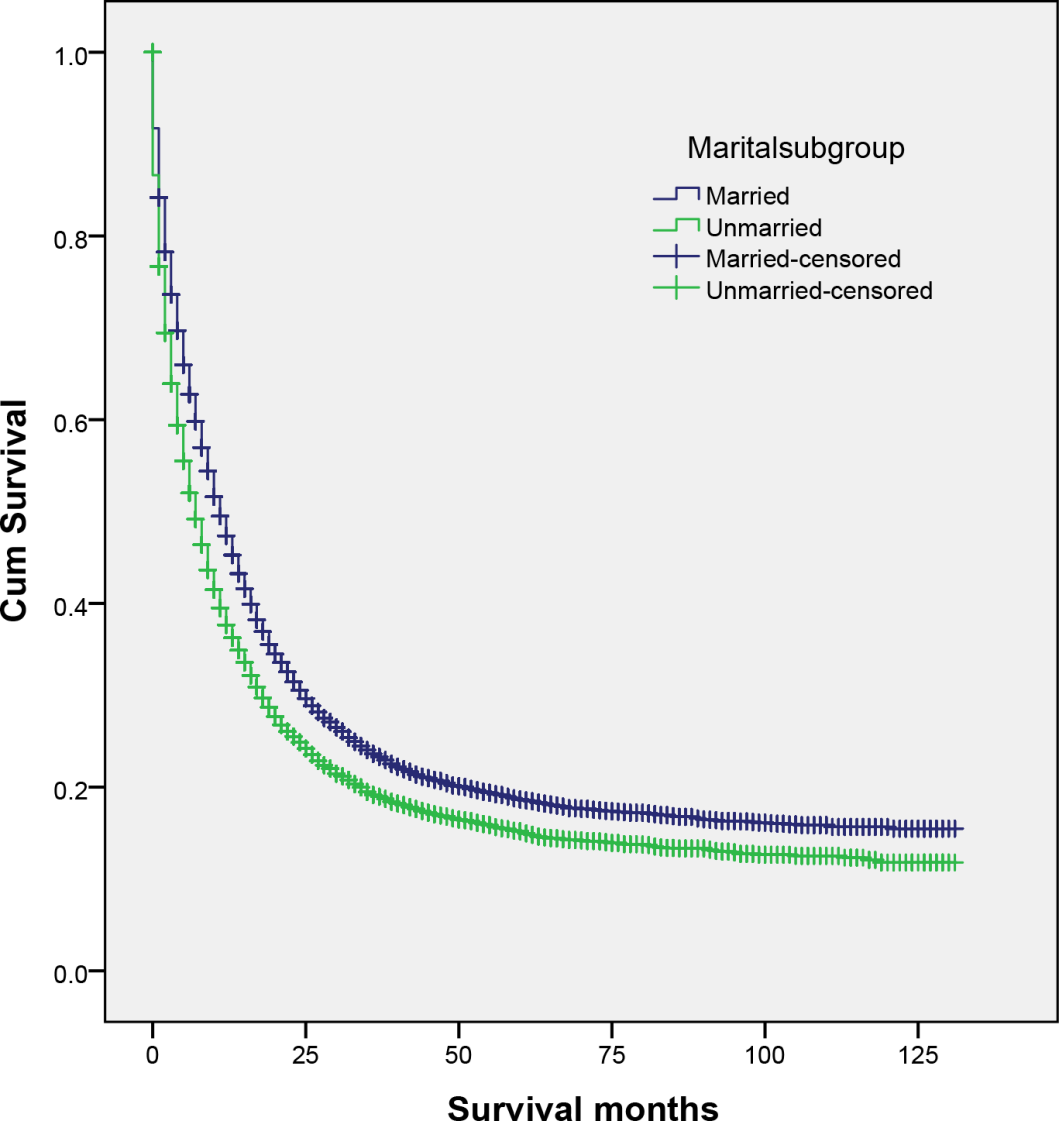
Survival curves for married and unmarried biliary tract cancer patients χ^2^ = 205.491, *P* < 0.001.

**Figure 2:**
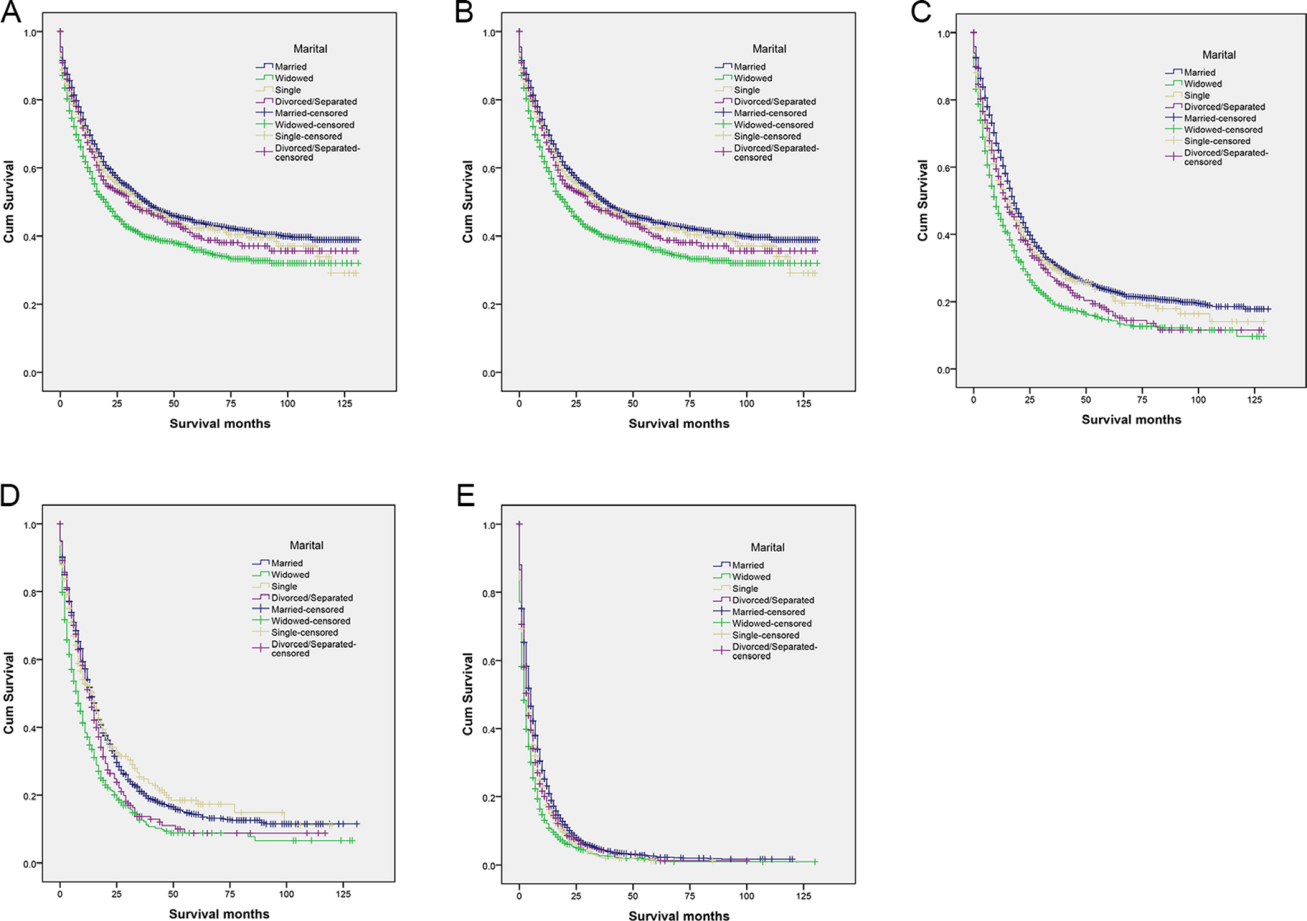
Survival curves for married and unmarried biliary tract cancer patients by stage (**A)** All stages: χ^2^ = 23225.337, *P* < 0.001 (**B)** Stage I: χ^2^ = 42.865, *P* < 0.001 (**C)** Stage II: χ^2^ = 92.511, *P* < 0.001 (**D**) Stage III: χ^2^ = 48.252, *P* < 0.001 (**E**) Stage IV: χ^2^ = 145.801, *P* < 0.001.

Married patients had higher 5-year CSS than unmarried patients (18.7% vs 15.2%) (*P* < 0.001) (Figure 1). Five-year CSS was 18.7% in the married group, 13.9% in the widowed group, 17.9% in the never married group, and 14.4% in the divorced/separated group; a univariate log rank test revealed that CSS differed significantly among all groups (*P* < 0.001) (Figure 2A). Additionally, elderly patients (*P* < 0.001), black patients (*P* < 0.001), patients with cholangiocarcinoma (*P* < 0.001), TNM stage III/IV (*P* < 0.001), poorly or undifferentiated tumors (*P* < 0.001), or advanced SEER stage (*P* < 0.001), and patients who did not undergo surgery (*P* < 0.001) were at higher risk of poor survival in a univariate analysis (Table 2).

**Table 2:** Univariate and multivariate survival analysis of associations between marital status and CSS in SEER database patients

		Univariate analysis		Multivariate analysis	
Variable	5-year CCS	Log rank χ^2^ test	*P*	HR (95% CI)	*P*
Sex		0.480	0.488		NI
Male	17.0%				
Female	17.2%				
Age		271.377	< 0.001		
≤60	20.9%			Reference	
>60	15.7%			1.388 (1.338–1.441)	< 0.001
Race		39.979	< 0.001		
White	16.9%			Reference	
Black	15.2%			1.049 (0.995–1.105)	0.77
Other^*^	19.7%			0.896 (0.856–0.938)	< 0.001
Tumor site		1197.390	< 0.001		
Gallbladder	19.6%			Reference	
Bile duct	10.5%			0.847 (0.815–0.881)	< 0.001
Ampulla of Vater	34.6%			0.569 (0.538–0.602)	< 0.001
AJCC 6th TNM stage		4995.646	< 0.001		
I	41.4%			Reference	
II	21.2%			1.553 (1.446–1.668)	< 0.001
III	13.3%			1.652 (1.525–1.789)	< 0.001
IV	2.0%			2.225 (2.007–2.466)	< 0.001
Unknown	8.1%			1.969 (1.816–2.135)	< 0.001
Grade		2945.362	< 0.001		
I	41.4%			Reference	
II	28.7%			1.275 (1.180–1.377)	< 0.001
III	15.6%			1.761 (1.630–1.902)	< 0.001
IV	17.1%			1.631 (1.372–1.939)	< 0.001
Unknown	7.4%			1.461 (1.353–1.577)	< 0.001
SEER Stage		3717.179	< 0.001		
Localized	34.9%			Reference	
Regional	21.1%			1.118 (1.047–1.193)	< 0.001
Distant	3.3%			1.212 (1.101–1.334)	< 0.001
Unknown	7.8%			1.000 (0.917–1.091)	< 0.001
Therapy		5505.595	< 0.001		
Surgery	33.3%			Reference	
No surgery	4.1%			2.448 (2.336–2.566)	< 0.001
Unknown	9.0%			1.962 (1.687–2.283)	< 0.001
Marital Status		284.788	< 0.001		
Married	18.7%			Reference	
Widowed	13.9%			1.245 (1.196–1.295)	< 0.001
Never married	17.9%			1.142 (1.090–1.197)	< 0.001
Divorced/Separated	14.4%			1.135 (1.077–1.196)	< 0.001

^*^ Other includes American Indian/Alaska native, Asian/Pacific Islander, and unknown. NI: not included in the multivariate survival analysis.

The following eight variables were identified as independent prognostic factors in multivariate analysis with Cox regression: age (> 60 years, hazard ratio (HR) 1.388, 95% confidence interval (CI) 1.338–1.441), race (other, HR 0.896, 95% CI 0.856–0.938), tumor site (bile duct, HR 0.847, 95% CI 0.815–0.881; ampulla of Vater, HR 0.569, 95% CI 0.538–0.602), TNM stage (stage II, HR 1.553, 95% CI 1.446–1.668; stage III, HR 1.652, 95% CI 1.525-1.789; stage IV, HR 2.225, 95% CI 2.007–2.466), pathological grade (grade II, HR 1.275, 95% CI 1.180–1.377; grade III, HR 1.761, 95% CI 1.630–1.902; grade IV, HR 1.631, 95% CI 1.372–1.939), SEER stage (regional, HR 1.118, 95% CI 1.047–1.193; distant, HR 1.212, 95% CI 1.101–1.334), therapy (no surgery, HR 2.448, 95% CI 2.336–2.566), and marital status (widowed, HR 1.245, 95% CI 1.196–1.295; never married, HR 1.142, 95% CI 1.090-1.197; divorced/separated, HR 1.135, 95% CI 1.077–1.196).

### Subgroup analysis of associations between marital status and survival for different TNM stages

We then analyzed the relationship between marital status and survival at each TNM stage. Marital status was an independent prognostic factor in each tumor stage both in univariate and multivariate analyses (*P* < 0.05). In addition, widowed patients in the localized stage group had lower survival rates than all other patients. Five-year CSS was reduced by 7.9% in stage I (35.9% vs 43.8%, *P* < 0.001), 8.9% in stage II (14.6% vs 23.5%, *P* < 0.001), 5.5% in stage III (8.8% vs 14.3%, *P* < 0.001), and 1.2% in stage IV (1.2% vs 2.4%, *P* < 0.001) widowed patients compared to married patients with the same stages. Finally, 5-year CSS rates did not differ between never married and married patients with either regional or distant stage disease (Table 3, Figure 2B–2D).

**Table 3:** Univariate and multivariate analysis of associations between marital status and CSS for different cancer stages

		Univariate analysis		Multivariate analysis	
AJCC 6th TNM stage/Variable	5-year CCS	Log rank χ^2^ test	*P*	HR (95% CI)	*P*
**Stage I**		42.865	< 0.001		
**Marital status**					< 0.001
Married	43.8%			Reference	
Widowed	35.9%			1.369 (1.244–1.506)	< 0.001
Never married	42.4%			1.089 (0.962–1.234)	0.178
Divorced/Separated	39.9%			1.119 (0.971–1.290)	0.119
**Stage II**		92.511	< 0.001		
**Marital status**					0.001
Married	23.5%			Reference	
Widowed	14.6%			1.522 (1.393–1.664)	< 0.001
Never married	23.7%			1.153 (1.037–1.282)	0.009
Divorced/Separated	17.1%			1.213 (1.080–1.363)	0.001
**Stage III**		48.252	< 0.001		
**Marital status**					< 0.001
Married	14.3%			Reference	
Widowed	8.8%			1.504 (1.330–1.701)	< 0.001
Never married	18.4%			0.983 (0.850–1.136)	0.813
Divorced/Separated	8.8%			1.152 (0.996–1.332)	0.056
**Stage IV**		145.801	< 0.001		
**Marital status**					< 0.001
Married	2.4%			Reference	
Widowed	1.2%			1.447 (1.355–1.545)	< 0.001
Never married	1.2%			1.172 (1.090–1.260)	< 0.001
Divorced/Separated	2.0%			1.144 (1.054–1.242)	0.001

### Subgroup analysis of associations between marital status and survival for different tumor sites

We also analyzed the association between marital status and survival for each tumor site. Marital status was again predictive of CSS in univariate and multivariate analyses (*p* < 0.05). CSS rates were higher in Ampulla of Vater cancer patients than in gallbladder cancer and cholangiocarcinoma patients. Widowed individuals still had the lowest survival rate among all patients (Table 4).

**Table 4:** Univariate and multivariate analysis of associations between marital status and CSS for different tumor sites

		Univariate analysis		Multivariate analysis	
Tumor site/Variable	5-year CCS	Log rank χ^2^ test	*P*	HR (95% CI)	*P*
**Gallbladder**		32.220	< 0.001		
**Marital status**					< 0.001
Married	20.4%			Reference	
Widowed	18.6%			1.195 (1.119–1.276)	< 0.001
Never married	19.4%			1.120 (1.033–1.215)	0.006
Divorced/Separated	18.6%			1.075 (0.981–1.178)	0.122
**Bile duct**		257.23	< 0.001		
**Marital status**					< 0.001
Married	12.0%			Reference	
Widowed	7.1%			1.480 (1.406–1.558)	< 0.001
Never married	11.4%			1.137 (1.069–1.209)	< 0.001
Divorced/Separated	7.1%			1.247 (1.165–1.335)	< 0.001
**Ampulla of Vater**		60.189	< 0.001		
**Marital status**					< 0.001
Married	37.2%			Reference	
Widowed	25.7%			1.574 (1.399–1.770)	< 0.001
Never married	36.8%			1.075 (0.934–1.239)	0.313
Divorced/Separated	31.5%			1.108 (0.942–1.305)	0.216

## DISCUSSION

In this study, we investigated associations between marriage and CSS in a large BTC patient population. We found that married patients have better cause-specific survival outcomes than unmarried patients, which includes widowed and separated/divorced patients and those who never married. This association between being married and better survival persisted even after adjusting for age, race, tumor site, pathology grade, TNM stage, SEER stage, and therapy in multivariable analyses. Moreover, widowed patients had a higher risk of cause-specific death than all other patient groups. Further subgroup analyses based on TNM stage and tumor site confirmed the prognostic value of marital status in BTC patients.

Previous studies have suggested that poorer prognoses in unmarried individuals may be due to delayed diagnosis at more advanced tumor stages in these patients [13, 19, 20]. However, in our patient population, the percentages of patients at each tumor stage were comparable among the four marriage status subgroups. Moreover, a higher proportion of widowed group patients (27.5%) had localized stage disease compared to married (21.6%), never married (22.6%), and divorced/separated (21.6%) group patients. These results suggest that delayed diagnosis cannot explain the poorer survival outcomes observed in widowed patients.

The exact mechanisms underlying the prognostic impact of marital status in GBC are unclear. Several biological, psychological, and social theories have been postulated to explain this phenomenon. It is well known that a diagnosis of cancer is psychologically distressing for most patients [21]. Because they do not have spouses to share their emotional burdens and contribute to their social support networks, unmarried cancer patients may experience more distress, depression, and anxiety than married patients [22, 23]. Additionally, marital status may affect adherence to prescribed treatments. Compared to unmarried patients, married patients are more likely to comply with treatment, to seek treatment at more prestigious centers, and to accept more aggressive treatment, all of which may contribute to better cancer control [24, 25].

Physiological changes accompanying stress and depression may worsen cancer outcomes through different mechanisms. For example, decreased psychosocial support and increased psychological stress result in immune dysfunction and contribute to tumor progression and mortality [26–28]. Furthermore, perceived lack of social support can reduce the activity of natural killer cells [29], resulting in dysregulation of various endocrine hormones, such as cortisol and catecholamines [26, 28]. Several studies demonstrate that cortisol and catecholamines can accelerate cancer growth and metastasis via immunosuppressive actions [30–32]. Cortisol activity has been identified as a prognostic factor in breast and lung cancer [32, 33]. Depression and low quality of life are also associated with increased production of VEGF, which may stimulate endothelial cell migration, proliferation, and proteolytic activity [34]. DiMatteo *et al*. found a strong association between depression and medical noncompliance [35], and women with depression who are diagnosed with breast cancer undergo definitive treatment less often and have worse survival outcomes [36].

Some limitations of this study should be considered when interpreting the results. First, the SEER database only provides marital status at the time of diagnosis and does not account for changes in marital status during the follow-up period, which may also influence outcomes. Second, the SEER database lacks details about the quality of marriages (e.g., satisfaction with the relationship, sexual activity), which might be associated with survival in GBC patients. For example, marital distress has long-term immune consequences and enhances the risk of a variety of health problems [37]. Third, and perhaps most important, the SEER database does not provide detailed data regarding chemotherapy, other types of therapy, subsequent therapy, comorbidities, recurrence, or socioeconomic factors.

Despite these potential limitations, our findings demonstrate that marital status is an independent prognostic factor for survival in patients with BTC. Specifically, unmarried patients are at greater risk of cancer-specific mortality. Psychosocial factors may be the main contributors to poorer survival outcomes in unmarried patients; additional social support should therefore be provided for these patients.
